# Case Report of Primary Splenic Angiosarcoma in a Young Patient: A Very Rare and Challenging Disease

**DOI:** 10.1155/cris/6763789

**Published:** 2026-04-15

**Authors:** Chakib Khoury, Christina Said, Maria Azzi, Emanuel-Youssef Dib, Mohamad Othman, Omar Tabbikha, Fadia Elias, Raja Wakim

**Affiliations:** ^1^ Department of Biomedical Sciences, Faculty of Medicine and Medical Sciences, University of Balamand, Kalhat, Lebanon, balamand.edu.lb; ^2^ Department of General Surgery, Faculty of Medicine, American University of Beirut, Beirut, Lebanon, aub.edu.lb; ^3^ Department of Hematology and Oncology, Mount Lebanon Hospital, Beirut, Lebanon; ^4^ Department of General Surgery, Mount Lebanon Hospital, Beirut, Lebanon

**Keywords:** angiosarcoma, sarcoma, splenectomy, splenic tumor, splenic angiosarcoma

## Abstract

**Introduction:**

Primary splenic angiosarcoma (PSA) is a rare type of angiosarcoma often associated with an unfavorable prognosis. The presenting symptoms are usually nonspecific, adding to the diagnostic difficulty of this disease. Limited cases have been described in younger adults, as most cases have been reported in individuals between 50 and 70 years old.

**Presentation of Case:**

We report the case of a 25‐year‐old patient with cerebral palsy who initially presented for high‐grade fever and left upper quadrant (LUQ) pain. On computed tomography (CT) scan, a large heterogenous mass was discovered in the spleen, and the patient underwent total splenectomy with pathological analysis confirming the diagnosis of PSA. The patient had no further complications and is still clinically stable.

**Discussion:**

PSA’s pathogenesis is not fully understood, though potential risk factors include exposure to chemicals or radiation, none of which were present in this case. Diagnosing PSA is challenging due to nonspecific symptoms and imaging findings that overlap with benign splenic lesions. Treatment primarily involves splenectomy, but prognosis remains poor due to the high likelihood of early metastasis. Adjuvant therapies, including chemotherapy and targeted agents, are under investigation.

**Conclusion:**

PSA is a rare and aggressive malignancy with significant diagnostic and therapeutic challenges. Early recognition and timely management may be important in selected cases, and outcomes remain poor, highlighting the need for further research to optimize treatment strategies.

## 1. Introduction

Angiosarcoma is a rare and aggressive type of malignancy arising originally from the endothelial cells lining blood vessels. It most commonly occurs as cutaneous lesions of the head and neck, but can also present in soft tissues, visceral organs, and bones. While angiosarcomas account for only 1% of all sarcomas, primary splenic angiosarcoma (PSA) is an exceedingly rare subset within this already uncommon group [1]. In other words, PSA represents an estimated annual incidence of scarcely one case in 4 million patients [2].

This unusual site for primary angiosarcoma is typically associated with poor prognosis due to its aggressive nature and potential for early metastasis, affecting 69%–100% of documented cases [1]. Thus, the mortality rate of PSA is as high as 27%, with an overall survival estimated to be only 12 months after diagnosis [2]. Most cases of PSA have been reported in individuals between 50 and 70 years old. However, limited cases have been described in younger adults.

The rarity of this diagnosis, with less than 300 documented cases of PSA, has limited the availability of detailed guidelines and large‐scale studies discussing the clinical course and treatment outcomes. Thus, case studies have tremendously helped guide patient care by providing valuable insight into diagnostic challenges and treatment options [3].

This case study intends to contribute to the existing literature through the presentation of a comprehensive account of a rare case of PSA diagnosed in a young patient. We will discuss the clinical presentation, diagnostic approach, and treatment employed, while highlighting the challenges associated with managing this rare and aggressive cancer.

## 2. Case Presentation

This is the case of a 25‐year‐old male patient known to have cerebral palsy since infancy, presenting for several days of prolonged high‐grade fever exceeding 38.5°C and left upper quadrant (LUQ) pain. The source of history is the patient’s parents, as he is deaf and mute. The parents denied any other symptoms, including diarrhea, vomiting, severe weight loss, nausea, or change in behavior from baseline. They also denied any recent exposure to a sick person or travel history.

**Figure 1:**
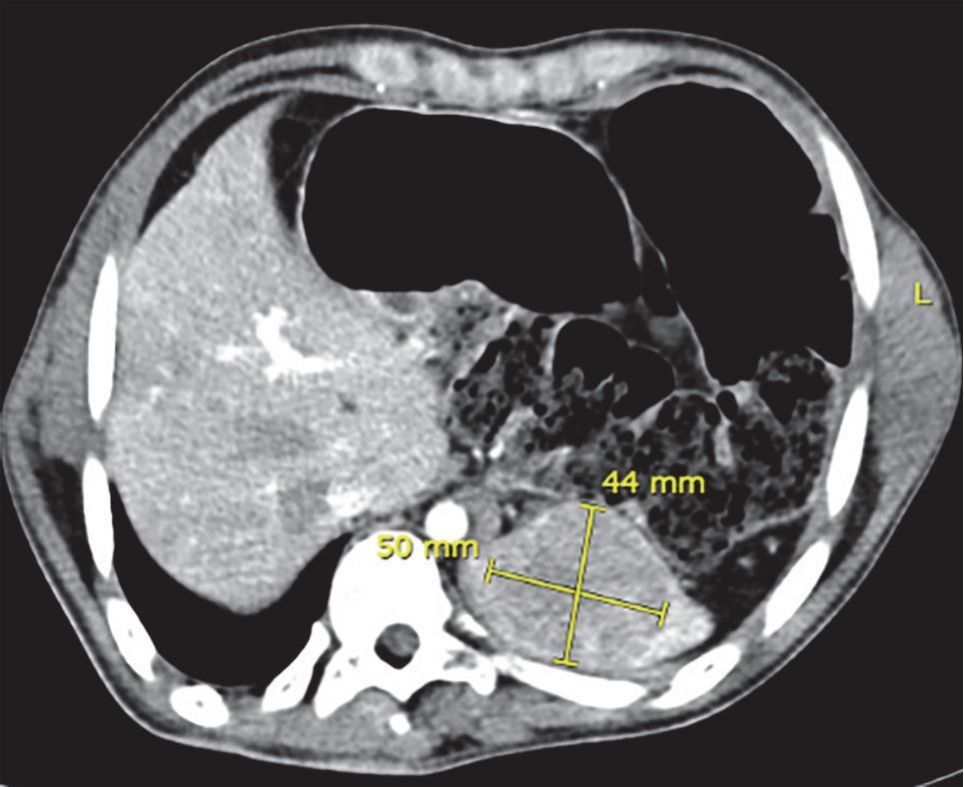
Computed tomography axial section of upper abdomen showing splenic mass measuring > 4.5 cm.

Physical examination showed an alert young man with cerebral palsy, no pertinent findings except those related to his condition (extremity posture). Laboratory tests were within normal range. A computed tomography (CT) of the abdomen and pelvis with intravenous contrast found a normal size spleen with numerous hypodense lesions, the largest showing heterogeneous enhanced parenchyma measuring 5 cm × 4.2 cm in the upper pole, suspecting hemangioma (Figure 1).

**Figure 2:**
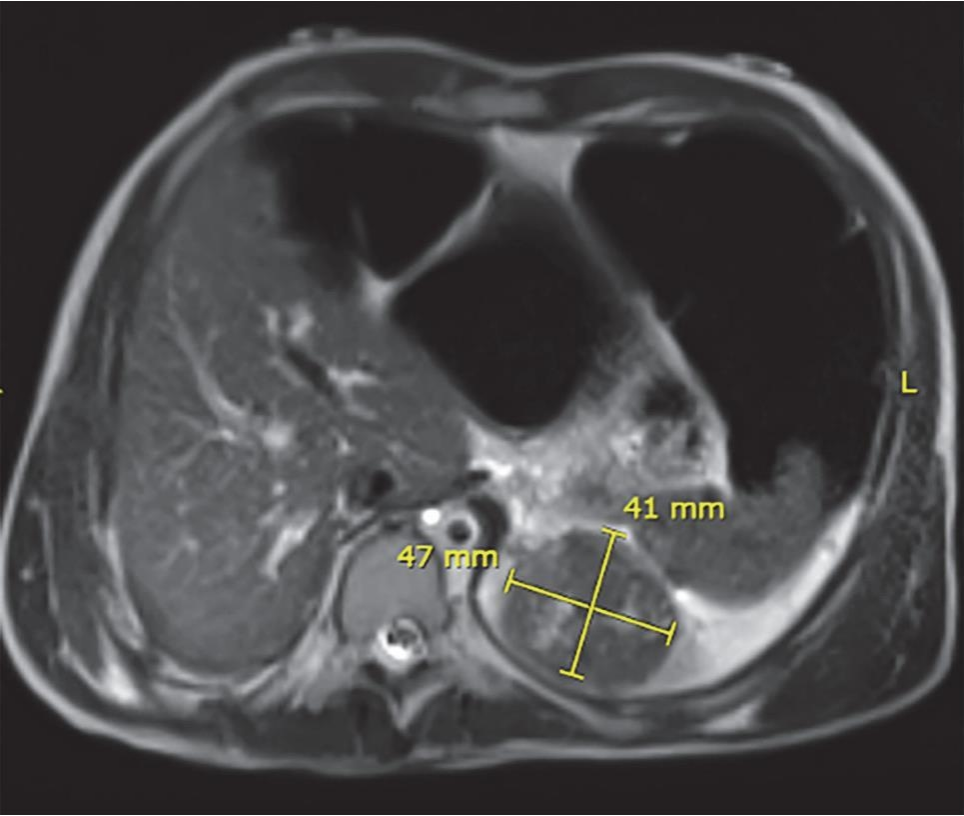
Magnetic resonance image in axial section showing the same splenic mass measuring over 4.5 cm.

A follow‐up magnetic resonance image (MRI) showed a 4.8 cm × 4.2 cm nodule in the superior medial aspect of the spleen with central linear high signal and no evidence of diffusion restriction, showing faint enhancement (Figure 2). A definitive diagnosis could not be identified. Based on the location of the tumor, no specific markers were ordered, and an endoscopic ultrasound was ruled out from the workup list as they will not provide any additional diagnostic value.

Assessment included contrast‐enhanced CT of the chest, abdomen, and pelvis, which showed no evidence of distant metastasis. Differential diagnoses included benign vascular lesions, lymphoma, inflammatory processes, and malignant tumor. Due to the inability to perform a percutaneous biopsy, a laparoscopic resection of the tumor was performed with no complications. Patient was discharged 4 days later. Histopathological analysis of the spleen yielded findings consistent with angiosarcoma, including strong positivity for cluster of differentiation (CD) −31, CD34, and erythroblast transformation‐specific‐related gene (ERG), confirming endothelial differentiation. Ki‐67 proliferation index was elevated at 56%.

On follow‐up 20‐months post discharge, the patient was in good health and reported no recurrence of symptoms, and follow‐up CT imaging of the abdomen and pelvis showed no significant findings. A longer follow‐up is required, given the known potential for delayed recurrence and metastasis.

We have obtained informed consent from the patient and his family.

## 3. Case Discussion

SAs’ pathogenesis is not completely well understood; Lui et al. [4] indicated that hemangiosarcoma formation begins from endothelial progenitor cells (EPCs) that express CD117, CD45, and CD34.

Possible associated factors to the development of PSA include arsenic, vinyl chloride, thorium dioxide, and ionizing radiation therapy [5, 6]. A case has been described following chemotherapy for a follicular lymphoma patient, but no clear relationship has been described yet [7]. None of these factors was present in our case. Previous case reports postulate that an SA can develop from an original hemangioma [8, 9].

A review of 122 articles from 1946 to 2021 found no gender predominance of PSA, and a mean age of 56.3 years, ranging from 19 to 89 years old [10]. There is a gap in the literature on the exact incidence rate of PSA, yet it is estimated to be around 0.14–0.23 cases per 1 million patients, and even less common in the pediatric age group. Males and females both usually present at older ages, 53 years and 68 years, respectively [2]. To our knowledge, this represents one of the youngest reported cases in the indexed literature.

With less than 300 cases in the literature describing SA presentation, it is very difficult to find a comprehensive and accurate diagnostic algorithm. SA has a wide variability of patient presentation, ranging from nonspecific generalized symptoms like dyspnea, weight loss, fatigue, and loss of appetite to LUQ pain, splenomegaly, and abdominal distention [10]. Splenomegaly is the most common finding (82%), and the most common symptom is abdominal pain (70.2%), with greater than half of these localized to the left abdominal quadrant (55.6%) [6, 10]. Laboratory abnormalities such as normocytic normochromic anemia, thrombocytopenia, and pancytopenia have been reported [6]. Our patient presented with persistent fevers unresponsive to antipyretics and nonspecific LUQ abdominal pain. The laboratory panels were within normal ranges.

Radiological diagnosis of PSA is challenging due to the lack of specific characteristics that distinguish it from other benign splenic tumors, such as benign hemangiomas or malignant Littoral cell sarcoma, to name a few. The two most commonly used modalities for diagnosis are CT scan and MRI [10]. On precontrast CT scans, the mass typically appears ill‐defined, with hyperdense areas indicating hemorrhage, hemosiderin deposits or spontaneous rupture into the peritoneal cavity. Additionally, precontrast images may show punctate and massive radial calcifications in some patients. Postcontrast images often exhibit heterogeneous enhancement, with less enhanced areas due to necrotic degeneration [11].

MRI is crucial for evaluating SAs as it reflects hemorrhage within the tumor. The signal intensity on T1‐ and T2‐weighted images varies from low to high, depending on the presence of necrosis and blood products. Contrast‐enhanced MR images reveal heterogeneous enhancement due to solid and necrotic areas. On DW‐MRI, the tumor exhibits low ADC values, which are indicative of a malignant lesion [12].

These imaging findings, however, can also be seen in benign hemangiomas, lymphomas, and metastasis, leading to potential diagnostic delay. That is why it is crucial for radiologists to be aware of this rare neoplasm and to consider it in the differential diagnosis when encountering a heterogeneously enhancing splenic mass to prevent such delays [12].

Finally, Barat et al.’s [13] review highlights the importance of [14] FDG‐PET/CT use in characterizing and differentiating benign from malignant solid focal splenic lesions, suggesting that FDG‐avid splenic lesions are likely malignant angiosarcomas [12].

Due to the rarity of PSA, the treatment is particularly challenging, and no standardized guidelines have been developed. However, localized SA is usually treated with total splenectomy. But given the infiltrative nature of the disease, attaining clear surgical margins early enough in the disease course is quite rare. In case of local invasion, debulking surgery can be used with resection of adjacent structures to improve prognosis and alleviate symptoms. Moreover, splenectomy done prior to rupture of the spleen has been correlated with an increased length of survival compared with splenectomy postrupture, as shown with a mean survival time of 14.4 months compared to 4.4 months, respectively [15]. However, this difference may reflect more advanced disease at presentation in rupture cases rather than a direct causal effect of early surgery.

Even though there was no statistically significant correlation between survival and nonsurgical approaches [16], chemotherapy and radiotherapy have been used as adjuvant or even neoadjuvant treatments to try and reduce the tumor burden. The most used chemotherapeutic regimens may include doxorubicin, ifosfamide, paclitaxel, gemcitabine, or docetaxel [17]. Most recently, targeted therapy, such as bevacizumab, has shown promising results in some cases of angiosarcoma [1]. These agents act as recombinant anti‐angiogenic antibodies and work by inhibiting VEGF receptors that are highly activated in angiosarcomas. Immunotherapy, such as checkpoint inhibitors and rIL2 (recombinant interleukin‐2) are also being explored in clinical studies for different types of angiosarcomas [18]. Evidence supporting systemic therapies remains limited to small case series and reports, with no randomized controlled trials demonstrating a clear survival benefit.

Most studies covering PSAs estimate median survival of around 5–12 months, with nearly all patient mortality being less than 3 years [6, 14]. Another study by Neuhauser et al. [16] on 28 cases reported a mean survival of 11 months; notably, a patient treated with chemotherapy and radiotherapy had residual disease at 8 years, while another patient treated with splenectomy alone had no residual disease at 9 years of follow‐up.

The nature of such a neoplasm dictates its dismal prognosis: highly proliferating anaplastic cells, which means high rates of local recurrence and systemic metastasis. Metastasis of angiosarcoma is relatively frequent and early, with rates of spread reported from 70% to 85% or even to 100% in others. Common sites of metastatic disease are the liver (89%), lung (78%), lymph nodes (56%), bone (22%), and less frequently brain and adrenals [2, 6]. A review by Mark et al. [19] showed that histological grading and appearance do not dictate clinical outcome: poorly‐differentiated lesions are likely to be as aggressive as well‐differentiated ones. Another analysis by Naka et al. [20] determined that tumor size and mitotic counts of malignant cells were significant prognostic factors. Follow‐up with regular imaging and blood tests is crucial to monitor disease progression and treatment‐associated complications.

This case underscores the importance of maintaining a high index of suspicion in indeterminate splenic lesions and emphasizes individualized multidisciplinary decision‐making.

## 4. Conclusion

PSA is a rare malignancy with a challenging diagnostic aspect. Treatment is based mainly on surgical removal, offering few options and limiting favorable patient outcomes. The management of SA remains complex and undefined, which highlights the need for multiple clinical trials and case studies to decipher the most optimal treatment plan.

## Funding

No funding was received for this manuscript.

## Consent

Written informed consent for publication of this case report and accompanying images was obtained from the patient’s mother acting as legal guardian, as the patient lacked decision‐making capacity to provide consent independently.

## Conflicts of Interest

The authors declare no conflicts of interest.

## Data Availability

The data that support the findings of this study are available from the corresponding author upon reasonable request.
